# Plant-Derived Cell-Free Biofactories for the Production of Secondary Metabolites

**DOI:** 10.3389/fpls.2021.794999

**Published:** 2022-01-28

**Authors:** Matthias Buntru, Nils Hahnengress, Alexander Croon, Stefan Schillberg

**Affiliations:** ^1^Fraunhofer Institute for Molecular Biology and Applied Ecology IME, Aachen, Germany; ^2^Institute of Phytopathology, Justus Liebig University, Giessen, Germany

**Keywords:** betanin, BYL, cell-free lysate, indigoidine, lycopene, metabolic enzymes

## Abstract

Cell-free expression systems enable the production of proteins and metabolites within a few hours or days. Removing the cellular context while maintaining the protein biosynthesis apparatus provides an open system that allows metabolic pathways to be installed and optimized by expressing different numbers and combinations of enzymes. This facilitates the synthesis of secondary metabolites that are difficult to produce in cell-based systems because they are toxic to the host cell or immediately converted into downstream products. Recently, we developed a cell-free lysate derived from tobacco BY-2 cell suspension cultures for the production of recombinant proteins. This system is remarkably productive, achieving yields of up to 3 mg/mL in a one-pot *in vitro* transcription–translation reaction and contains highly active energy and cofactor regeneration pathways. Here, we demonstrate for the first time that the BY-2 cell-free lysate also allows the efficient production of several classes of secondary metabolites. As case studies, we synthesized lycopene, indigoidine, betanin, and betaxanthins, which are useful in the food, cosmetic, textile, and pharmaceutical industries. Production was achieved by the co-expression of up to three metabolic enzymes. For all four products, we achieved medium to high yields. However, the yield of betanin (555 μg/mL) was outstanding, exceeding the level reported in yeast cells by a factor of more than 30. Our results show that the BY-2 cell-free lysate is suitable not only for the verification and optimization of metabolic pathways, but also for the efficient production of small to medium quantities of secondary metabolites.

## Introduction

Secondary metabolites are organic natural products synthesized by plants, microbes, or animals *via* enzymatic cascades. They are generally not required for normal growth and development and instead facilitate ecological interactions, although the benefit to the producer is not always clear. However, many secondary metabolites, including alkaloids, terpenoids, polyketides, antibiotics, peptides, and growth hormones, have structures or biological activities that make them useful for applications in the chemical, agri-food, cosmetic, and pharmaceutical industries. Traditionally, secondary metabolites are extracted from their natural sources using solvents or are produced by chemical synthesis. However, both methods tend to be inefficient and harmful to the environment, making them costly and unsustainable. Furthermore, many natural products are too complex for chemical synthesis. To overcome these drawbacks, the industrial-scale production of secondary metabolites is often achieved by the metabolic engineering of microbial cells. However, the preparation of production strains is laborious and the heterologous production of some secondary metabolites can be difficult or even impossible due to the lack of precursors, the low activity of the introduced enzymes, or the inhibition of cell growth by the product (Pyne et al., 2019; Zhang and Too, 2020).

Cell-free systems avoid many of the limitations of cell-based expression and are now widely used to optimize metabolic pathways and produce secondary metabolites (Li et al., 2018; Bogart et al., 2021). For example, diketopiperazine, limonene and various indole alkaloids have been produced in *Escherichia coli* lysates or the commercial *E. coli*-based PURExpress system (Goering et al., 2017; Dudley et al., 2020; Khatri et al., 2020). These open cell-free systems facilitate manipulation, monitoring, optimization, and sampling, providing the following advantages over cell-based systems: (i) simplified processes for the introduction and optimization of complex pathways, including complex multi-domain enzymes; (ii) simple adjustment of suitable reaction conditions; (iii) fast reaction rates; and (iv) tolerance of otherwise toxic products. The combination of these advantages can lead to a significant increase in product yields.

We recently developed a new cell-free expression platform based on tobacco BY-2 cells. With the exception of the vacuole and the nucleus, the cell-free BY-2 lysate contains the entire contents of the cell, including functional mitochondria, which provide the energy for protein biosynthesis and produces recombinant proteins with yields of up to 3 mg/mL in a coupled transcription–translation batch process (Buntru et al., 2015; Madduri et al., 2018; Schillberg et al., 2019). The productivity of the BY-2 lysate (BYL) is therefore in the same range as prokaryotic cell-free systems and about 15-fold higher than any commercial eukaryotic cell-free batch process. Numerous proteins that are difficult to produce in intact cells, including membrane proteins, plant antigens and transcription factors, have been produced successfully in the BYL system (Buntru et al., 2015; Havenith et al., 2017; Wu et al., 2019). Here, we describe for the first time the use of the BYL system for the production of secondary metabolites, using lycopene, indigoidine, and betalains as case studies.

**FIGURE 1:**
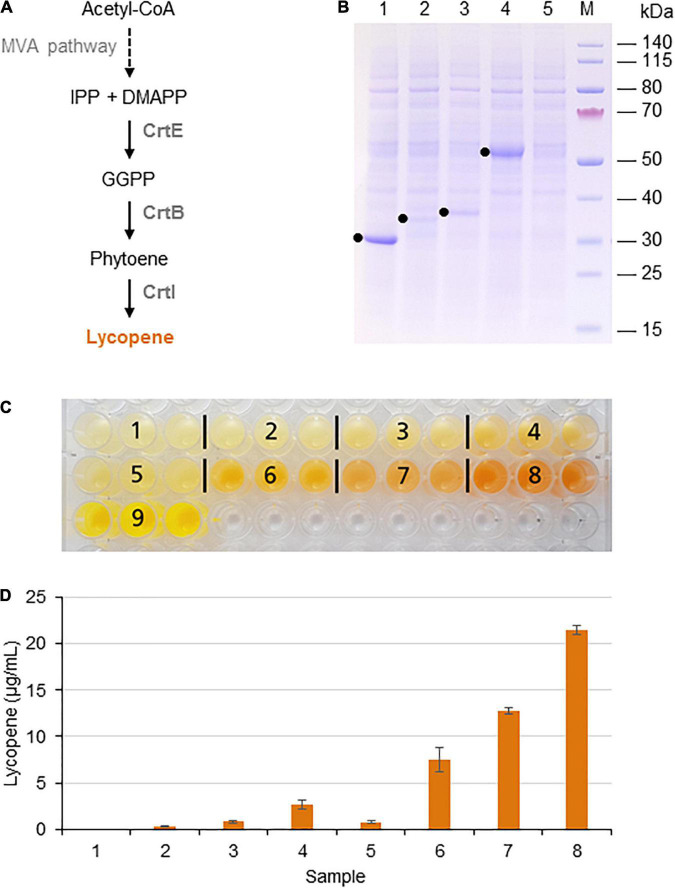
Lycopene biosynthesis in the BY-2 cell-free system. **(A)** Schematic overview of the endogenous mevalonate (MVA) pathway and the engineered carotenoid biosynthesis pathway in the BYL system. These pathways are localized in the cytosol or the cytosolic fraction of BYLs. Solid arrows represent single enzymatic steps and dashed arrows represent multiple enzymatic steps. The reactions catalyzed by *P. ananatis* CrtE, CrtB, and CrtI are indicated. IPP, isopentenyl diphosphate; DMAPP, dimethylallyl diphosphate; GGPP, geranylgeranyl diphosphate; CrtE, GGPP synthase; CrtB, phytoene synthase; and CrtI, phytoene desaturase. **(B)** SDS-PAGE analysis of BYL transcription–translation reactions after 44 h at 25°C and 70% humidity, shaking at 500 rpm. In each case, 0.5 μL of the reaction mix was loaded onto a 4–12% (w/v) gradient gel. Lane 1: Strep-eYFP (29 kDa), lane 2: CrtE-Strep (34 kDa), lane 3: CrtB-Strep (36 kDa), lane 4: CrtI-Strep (56 kDa), and lane 5: no template control. **(C)** Image of the *in vitro* reactions after incubation for 21 h: (1) no-template control, (2) CrtE, (3) CrtB, (4) CrtI, (5) CrtE + CrtB, (6) CrtB + CrtI, (7) CrtE + CrtI, (8) CrtE + CrtB + CrtI, and (9) Strep-eYFP. **(D)** Lycopene produced in BYL transcription–translation reactions after incubation for 21 h: (1) no-template control, (2) CrtE, (3) CrtB, (4) CrtI, (5) CrtE + CrtB, (6) CrtB + CrtI, (7) CrtE + CrtI, and (8) CrtE + CrtB + CrtI. After lycopene extraction, the concentration was determined by absorbance spectrophotometry at 472 nm and was compared to a standard curve prepared using commercial lycopene. Data represent the means and standard deviations of three independent transcription–translation experiments.

Lycopene is a bright red carotenoid found in tomatoes and other red fruits and vegetables. It is widely used as a natural food coloring, and its antioxidant activity has led to tests as a cancer therapeutic (Saini et al., 2020; Nowroozi et al., 2021). Lycopene is synthesized from the precursor isopentenyl diphosphate (IPP) and its isomer dimethylallyl diphosphate (DMAPP). DMAPP is then converted in three enzymatic steps to geranylgeranyl pyrophosphate (GGPP), phytoene, and finally lycopene (Figure 1A).

**FIGURE 2:**
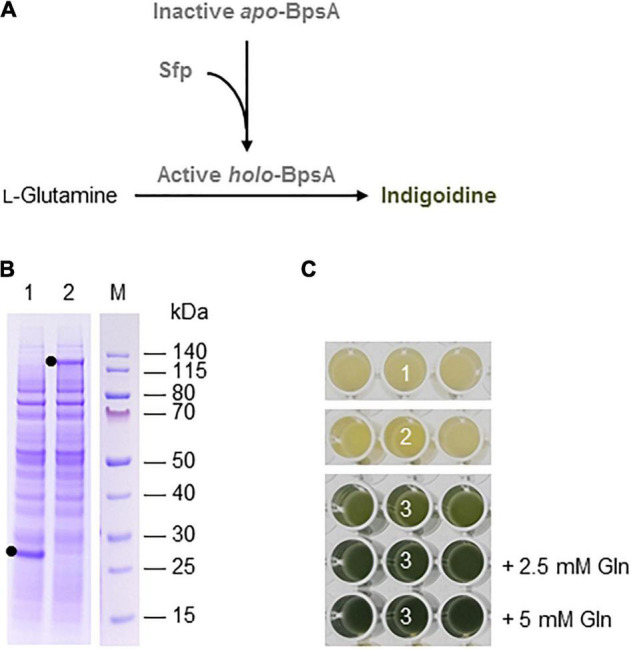
Indigoidine biosynthesis in the BY-2 cell-free system. **(A)** Schematic overview of the engineered indigoidine biosynthesis pathway. The inactive *apo*-form of blue pigment synthetase A (BpsA) from *S. lavendulae* is activated by the 4′-phosphopantetheinyl transferase Sfp from *B. subtilis* in a reaction requiring coenzyme A. The active *holo*-form of BpsA then catalyzes the conversion of two L-glutamine molecules to indigoidine. **(B)** SDS-PAGE analysis of BYL transcription–translation reactions after incubation for 44 h at 25°C and 70% humidity, shaking at 500 rpm. In each case, 0.5 μL of the reaction mix was loaded onto a 4–12% (w/v) gradient gel. Lane 1: Sfp (26 kDa), lane 2: BpsA (141 kDa). **(C)** Image of the *in vitro* reactions after incubation for 18 h: (1) Sfp, (2) BpsA, and (3) Sfp + BpsA. Reactions were supplemented with L-glutamine (Gln) as indicated.

Indigoidine is a non-ribosomal peptide (NRP) produced and excreted by some species of bacteria. It is a blue pigment that has been considered as a more sustainable alternative for industrial dyes (Wehrs et al., 2019), but it has also been evaluated for its antimicrobial activity (Cude et al., 2012; Gromek et al., 2016). Indigoidine is synthesized by the condensation of two molecules of L-glutamine in a reaction catalyzed by an NRP synthetase, which is activated by an Sfp-type phosphopantetheinyl transferase (Kim et al., 2018; Figure 2A).

**FIGURE 3:**
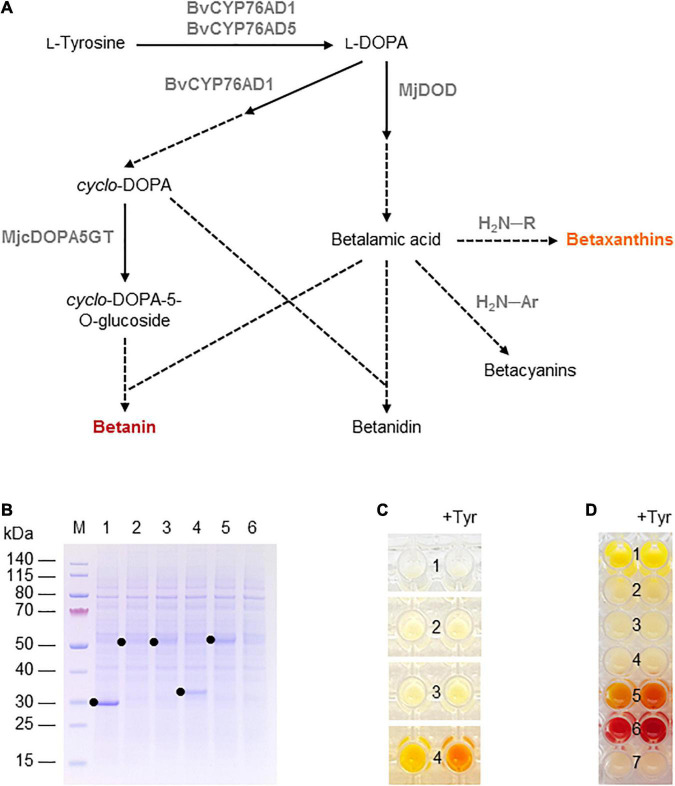
Betalain biosynthesis in the BY-2 cell-free system. **(A)** Schematic overview of the betalain biosynthesis pathway. Solid lines are enzymatic reactions and dashed lines are spontaneous reactions. R = any organic group; Ar = any organic aromatic group. **(B)** SDS-PAGE analysis of BYL transcription–translation reactions after incubation for 44 h at 25°C and 70% humidity, shaking at 500 rpm. In each case, 0.5 μL of the reaction mix was loaded onto a 4–12% (w/v) gradient gel. Lane 1: Strep-eYFP (29 kDa), lane 2: BvCYP76AD1_W13L (56 kDa), lane 3: BvCYP76AD5 (57 kDa), lane 4: MjDOD (30 kDa), lane 5: MjcDOPA5GT (57 kDa). **(C)** Image of the *in vitro* reactions for betaxanthin biosynthesis after incubation for 24 h: (1) no-template control, (2) BvCYP76AD5, (3) MjDOD, and (4) BvCYP76AD5 + MjDOD. Reactions were supplemented with 5 mM L-tyrosine (Tyr) as indicated. **(D)** Image of the *in vitro* reactions for betacyanin biosynthesis after incubation for 24 h: (1) Strep-eYFP, (2) BvCYP76AD1_W13L, (3) MjDOD, (4) MjcOPA5GT, (5) BvCYP76AD1_W13L + MjDOD, (6) BvCYP76AD1_W13L + MjDOD + MjcOPA5GT, and (7) no-template control. Reactions were supplemented with 5 mM L-tyrosine (Tyr) as indicated.

Betalains are pigments found in the flowers, fruits, and/or vegetative organs of many plants in the order Caryophyllales, as well as leaf fungi and bacteria (Contreras-Llano et al., 2019). They are structurally related to alkaloids and can be divided into the red-violet betacyanins (e.g., betanin) and the yellow-orange betaxanthins. Betalain biosynthesis in plants begins with the conversion of tyrosine into L-3,4-dihydroxyphenylalanine (L-DOPA) by a cytochrome P450 hydroxylase and then to betalamic acid by a DOPA 4,5-dioxygenase (DOD) enzyme. Betalamic acid then reacts with various amino acids or amines to form diverse yellow-orange betaxanthins. Alternatively, L-DOPA is oxidized by the diphenolase activity of a cytochrome P450 to dopaquinone, which spontaneously cyclizes into *cyclo*-DOPA. The latter is converted to *cyclo*-DOPA-5-O-glucoside by a glucosyltransferase and reacts with betalamic acid to form the red pigment betanin (Polturak and Aharoni, 2018; Figure 3A). Alternatively, the order of glucosylation and condensation can be reversed. The glucosylated betanin is dramatically more stable than the unstable intermediate betanidin. Betalains are used as food colorants, but also possess antimicrobial, antimalarial and antidiabetic properties leading to their assessment as drug candidates (Madadi et al., 2020). For example, betanin (the most common betacyanin) from red beet has been approved for use as a natural colorant in food and cosmetic products, and is also being evaluated for its anti-inflammatory and hepatoprotective activity *in vitro* (Vieira Teixeira da Silva et al., 2019).

Here, we report the successful expression of metabolic enzymes in the BYL system leading to the formation of three different classes of secondary metabolites: terpenes (lycopene), NRPs (indigoidine), and alkaloid-like betalains (betanin and betaxanthins). Given the inherent color of the products, their accumulation in the cell-free system can easily be monitored using non-invasive methods and the performance of individual enzymes can be determined by separate expression and step-by-step construction of the pathway. In addition, precursors can be added to the cell-free system to maximize product accumulation. This is the first step toward the wider use of the BYL platform for the engineering of metabolic pathways to produce valuable secondary metabolites.

## Materials and Methods

### Construction of Expression Vectors

All cDNAs were transferred to the expression vector pLenEx (Supplementary Figure S1A), which was fully synthesized by BioCat (Heidelberg, Germany). The vector backbone is based on the plasmid pIVEX_GAA_Omega (Buntru et al., 2015) with some modifications. The transcriptional start site GAAAGA upstream of the tobacco mosaic virus 5′ omega leader sequence was replaced with GGGAGA (Buntru et al., 2017). The partial *lacZ* sequence and the F1 origin of replication were removed. To construct the control vector pLenEx_Strep-eYFP, expressing the fluorescent protein eYFP including an N-terminal streptavidin affinity tag, the Strep-eYFP sequence was amplified by PCR using primers 5′-GGT AGT CCA TGG CTT GGT CTC ATC CGC AAT TC-3′ and 5′-GAC ACG GGT ACC TTA TTA CTT GTA CAG CTC GTC CAT GC-3′, with pIVEX_GAA_Omega_Strep-eYFP as the template (Buntru et al., 2015). The PCR product was digested with *Nco*I and *Kpn*I (underlined in the primer sequences) and inserted at the *Nco*I and *Kpn*I sites of pLenEx.

The DNA fragments encoding the full−length *Pantoea ananatis* CrtE, CrtB, and CrtI proteins (GenBank accession numbers WP_176017233, WP_013027995, and WP_176017230, respectively) for lycopene synthesis, the *Streptomyces lavendulae* BpsA and *Bacillus subtilis* Sfp proteins (GenBank accession numbers QYB25025 and WP_015715234, respectively) for indigoidine synthesis, and the *Beta vulgaris* CYP76AD1_W13L, *Mirabilis jalapa* DOD, *M. jalapa* cDOPA5GT and *B. vulgaris* CYP76AD5 proteins (GenBank accession numbers AET43290, BAG80686, BAD91803, and AJD87473, respectively) for betalain synthesis, were synthesized as double-stranded DNA fragments (Integrated DNA Technologies, Coralville, IA, United States) including overhangs suitable for Gibson assembly (Gibson et al., 2009). The double-stranded DNA fragments were introduced into pLenEx_Strep-eYFP at the *Nco*I and *Kpn*I sites by Gibson DNA assembly using NEBuilder HiFi DNA Assembly Master Mix (New England Biolabs, Frankfurt, Germany), replacing the Strep-eYFP cDNA with the sequence encoding the enzyme of interest (Supplementary Figure S1B). For CrtE, CrtB, and CrtI, a C-terminal streptavidin affinity tag sequence (StrepII tag) was included, and an additional alanine residue was introduced after the start codon to create an *Nco*I restriction site. The *bpsA* and *sfp* sequences were codon optimized for tobacco (*Nicotiana tabacum*) using the codon optimization tool provided by Integrated DNA Technologies, and the first codon after the start codon was replaced with glycine to create an *Nco*I restriction site as previously described (Wehrs et al., 2019). The activity of CYP76AD1 was increased by introducing a W13L mutation (DeLoache et al., 2015). For MjDOD and MjcDOPA5GT, an additional alanine was introduced after the start codon to create an *Nco*I restriction site. The integrity of all 10 final expression vectors (Table 1) was verified by DNA sequencing, confirming the coding sequence of each expression cassette and the connection to the untranslated regions.

**TABLE 1:** List of plasmids used in this work.

Expression vector	Target protein/enzyme and cDNA source	Pathway
pLenEx_Strep-eYFP	Enhanced yellow fluorescent protein from *Aequorea victoria*	–
pLenEx_crtE-Strep	Geranylgeranyl diphosphate synthase from *Pantoea ananatis*	Lycopene
pLenEx_crtB-Strep	Phytoene synthase from *P. ananatis*	Lycopene
pLenEx_crtI-Strep	Phytoene desaturase from *P. ananatis*	Lycopene
pLenEx_bpsA	Blue pigment synthetase A from *Streptomyces lavendulae*	Indigoidine
pLenEx_sfp	4′-Phosphopantetheinyl transferase from *Bacillus subtilis*	Indigoidine
pLenEx_CYP76AD1_W13L	Monophenolase/diphenolase from *Beta vulgaris*	Betalains
pLenEx_MjDOD	DOPA-4,5-dioxygenase from *Mirabilis jalapa*	Betalains
pLenEx_MjcDOPA5GT	*cyclo*-DOPA glucosyltransferase from *M. jalapa*	Betalains
pLenEx_CYP76AD5	Monophenolase from *Beta vulgaris*	Betalains

### Preparation of Tobacco Cell-Free Lysate and Reaction Mix

The tobacco cell-free lysate (BYL) was prepared as previously described (Madduri et al., 2018) with minor modifications. Tobacco BY-2 cells were cultivated batch-wise in shake flasks and were harvested during the exponential growth phase at a packed cell volume of 20–25% (v/v). They were treated with 3% (v/v) Rohament CL and 0.2% (v/v) Rohapect UF (AB Enzymes, Darmstadt, Germany) directly in the fermentation medium. The osmolarity was adjusted by adding 360 mM mannitol. The resulting protoplasts were layered onto a discontinuous Percoll gradient containing (from bottom to top) 70% (v/v, 3 mL), 40% (v/v, 5 mL), 30% (v/v, 3 mL), 15% (v/v, 3 mL), and 0% (3 mL) Percoll (GE Healthcare, Munich, Germany) in 0.7 M mannitol, 20 mM MgCl_2_, and 5 mM PIPES-KOH (pH 7.0) in a 50-mL polypropylene tube (Greiner Bio-One, Frickenhausen, Germany). After centrifugation at 6,800 × *g* for 1 h at 25°C in a swing-bucket rotor, evacuolated protoplasts were recovered from the 40–70% (v/v) Percoll solution interface and suspended in three volumes of TR buffer (30 mM HEPES-KOH (pH 7.4), 60 mM potassium glutamate, 0.5 mM magnesium glutamate, 2 mM DTT) supplemented with one tablet per 50 mL of Complete EDTA-free Protease Inhibitor Mixture (Roche Diagnostics, Mannheim, Germany). The protoplasts were disrupted on ice using 15 strokes of a Dounce homogenizer (Braun, Melsungen, Germany), and the nuclei and non-disrupted cells were removed by centrifugation at 500 × *g* for 10 min at 4°C. Standard cell-free reactions contained 40% (v/v) BYL, 20 mM HEPES-KOH (pH 7.8), 9 mM magnesium glutamate, 20 mM potassium glutamate, 4 mM ATP, 1.6 mM GTP, 1.6 mM CTP, 1.6 mM UTP, 100 μg/mL chloramphenicol, and 30 ng/μL in-house T7 RNA polymerase. The reaction mix was supplemented with 2.5% (w/v) trehalose as a cryoprotectant and then frozen in 1-mL aliquots at –80°C.

### Coupled Transcription–Translation Cell-Free Reaction

Coupled transcription–translation reactions were carried out in aliquots of 50, 150, or 500 μL at 25°C and 70% humidity, shaking at 500 rpm (12.5 mm amplitude) in a Lab-Therm LT-X incubator shaker (Kuhner, Herzogenrath, Germany). Ten to 30 ng/μL (5–10 nM) plasmid DNA was added to the cell-free reaction mix. BYL mixtures for betanin and indigoidine production were optionally supplemented at the start of the reaction with 5 mM tyrosine and 2.5 or 5 mM glutamine, respectively, as precursors.

### SDS-PAGE Analysis

Samples were separated by SDS-PAGE in precast NuPAGE 4–12% (w/v) polyacrylamide Bis-Tris gels (Thermo Fisher Scientific, Waltham, MA, United States) alongside PageRuler Prestained Protein Ladder markers (Thermo Fisher Scientific). The gels were stained with Coomassie Brilliant Blue R-250.

### Metabolite Detection

Lycopene was extracted as described by Dzakovich et al. (2019) with modifications. Briefly, 150 μL of the BYL sample was mixed sequentially with 200 μL methanol and 600 μL hexane/acetone (1:1) by vortexing. We then added 300 μL water to induce phase separation, vortexed the mixture briefly and centrifuged at 5,000 × *g* for 30 s. The upper organic phase was transferred to a fresh microcentrifuge tube and the absorbance was measured at 472 nm (Shi and Le Maguer, 2000) using quartz cuvettes and an Infinite M200 reader (Tecan, Männedorf, Switzerland). The quantity of lycopene was determined by generating a standard curve based on different concentrations of a commercial lycopene standard (Sigma-Aldrich, Seelze, Hanover, Germany) in the organic phase prepared from BYL reactions without a DNA template.

Indigoidine was detected by optical verification of a color change in the BYL reaction samples from pale yellow to blue-green.

For betanin detection, insoluble compounds were removed by passing the samples through a regenerated cellulose 0.2-μm filter (Sartorius, Göttingen, Germany). The samples were then analyzed by chromatography using an Ultimate3000 UHPLC system (Thermo Fisher Scientific) including a quaternary pump, autosampler, column oven and UV-detector. The sampler temperature was set to 10°C and the column compartment to 25°C. Compounds of interest were analyzed in reversed phase mode on a LiChrosorb RP-18 column (250 × 4.6 mm, 5 μm) (Merck, Darmstadt, Germany). We separated 5.0-μL samples at a flow rate of 0.5 mL/min using 5.0% (v/v) methanol plus 94.9% (v/v) water with 0.1% (v/v) trifluoroacetic acid as eluent A and 95.0% (v/v) methanol plus 4.9% (v/v) water with 0.1% (v/v) trifluoroacetic acid as eluent B. The program began with an isocratic segment at 100% eluent A for 5 min, followed by a linear gradient to 60.0% eluent B (5–17 min), a linear gradient to 100% eluent B (17–19 min), an isocratic segment at 100% eluent B (19–23 min) and finally a linear gradient to 100% eluent A (23–24 min). The column was re-equilibrated for 6 min in 100% eluent A. Betanin was detected at 536 nm and was compared to a commercial betanin standard (Sigma-Aldrich). The formation of betaxanthins was detected by optical verification of a color change in the BYL reaction samples from pale yellow to bright yellow to orange.

## Results

### Expression Constructs

All target cDNAs were cloned separately into the expression vector pLenEx for the cell-free production of lycopene (three genes), indigoidine (two genes), and betalains (four genes) (Supplementary Figure S1B). Codon optimization of the target cDNA is usually not required to improve production in the cell-free BYL lysate. However, the bacterial *bpsA* and *sfp* sequences were codon optimized for tobacco to facilitate comparison with a previous study in which codon-optimized sequences were used for cell-based production in *Rhodosporidium toruloides* (Wehrs et al., 2019). In the case of *bpsA*, we also removed sequence repeats, hairpins and regions with a high GC content (>70%) to ensure efficient cDNA synthesis. For similar reasons, a C-terminal StrepII tag sequence was introduced into the CrtE, CrtB, and CrtI constructs since Matthäus et al. (2014) have shown that this tag did not interfere with the catalytic activity of the heterologous enzymes. We also expressed the fluorescent marker eYFP, which usually accumulates to levels of 3 mg per mL cell-free reaction (Schillberg et al., 2019), as a positive control to ensure cell-free protein synthesis was working as anticipated.

All 10 target genes were expressed under the control of the T7 promoter in the pLenEx expression vector. The T7 polymerase and all other factors necessary for *in vitro* transcription and translation, such as nucleoside triphosphates (NTPs), amino acids and salts, are already provided in the lysate. Accordingly, only vectors carrying the appropriate cDNAs need to be added to the cell-free lysate to facilitate rapid protein biosynthesis, enzyme analysis and the accumulation of secondary metabolites in a single reaction vessel.

### Lycopene Biosynthesis

Lycopene biosynthesis was achieved by expressing the enzymes CrtE, CrtB, and CrtI from the bacterium *P. ananatis*. These enzymes convert IPP and its isomer DMAPP to lycopene in three steps (Figure 1A). First, the three target proteins were produced separately in batch reactions, using the same molar concentration (5 nM) in each case to account for differences in cDNA size. The plasmid concentration of 5 nM was selected based on the optimal concentration of the template plasmid pLenEx_Strep-eYFP for Strep-eYFP production in the BYL system. After 44 h, BYL aliquots were analyzed by SDS-PAGE. CrtI accumulated to high levels, similar to the control protein eYFP (Figure 1B). In contrast, the levels of CrtE and CrtB were significantly lower, as shown by the weak bands barely visible against the background of tobacco host proteins. Nevertheless, the bands representing all three proteins were of the correct size: 34 kDa (CrtE), 36 kDa (CrtB), and 56 kDa (CrtI).

Next, we expressed the three enzymes individually, in pairs and also all three simultaneously in a single batch reaction. As above, we accounted for differences in cDNA size by using the same molar concentration of each construct (5 nM) in the individual reactions. In the combinatorial reactions we used equimolar concentrations of each construct and a total molar concentration of 5 nM. After 21 h, the reaction with three plasmids producing the full complement of enzymes (CrtE, CrtB, and CrtI) showed the most intense orange color, indicating the accumulation of large amounts of lycopene (Figure 1C). In contrast, there was no significant color in any of the single or double reactions lacking CrtI, which is understandable because this enzyme catalyzes the final step in the pathway. However, an orange color was observed in the reactions containing CrtI alone or paired with one of the other enzymes, although it was not as intense as the reaction with all three enzymes. The visual observations were confirmed by extracting the lycopene and measuring its absorbance at 472 nm. As expected, the highest level of 21 μg per mL cell-free reaction was detected when expressing all three enzymes (Figure 1D). However, significant levels were also detected when expressing CrtI alone (3 μg/mL) or in combination with CrtB (7 μg/mL) or CrtE (13 μg/mL), indicating that the precursors IPP/DMAPP, GGPP, and/or phytoene are present in the cell-free lysate. Carotenoids are found in all higher plants and related enzymes are also present in the proplastids of tobacco BY-2 cells (Baginsky et al., 2004).

### Indigoidine Biosynthesis

Indigoidine biosynthesis was achieved by expressing the *S. lavendulae* blue pigment synthetase A (*bpsA*) and *B. subtilis* 4′-phosphopantetheinyl transferase (*sfp*) genes. Following the activation of BpsA by the Sfp transferase, BpsA catalyzes the condensation of two molecules of L-glutamine to form indigoidine (Figure 2A). As above, we initially expressed the enzymes separately for 44 h using the same molar concentration of each construct (5 nM). SDS-PAGE analysis confirmed the presence of each protein, with anticipated band sizes of 141 and 26 kDa for BpsA and Sfp, respectively (Figure 2B).

Next we produced BpsA and Sfp alone using 5 nM of each construct and simultaneously using equimolar concentrations of each construct (2.5 nM). Indigoidine formation was detected optically based on a color change from pale yellow to blue-green. As expected, the expression of Sfp alone did not change the color of the lysate (number 1 in Figure 2C). The expression of BpsA alone led to a very slight color change of the BYL reaction samples (number 2 in Figure 2C). BpsA was probably activated by endogenous phosphopantetheinyl transferases with broad substrate spectrum (Yalpani et al., 2001), albeit with very low efficiency. The co-expression of both enzymes confirmed that the NRP synthetase BpsA was efficiently activated by the phosphopantetheinyl transferase Sfp, leading to the accumulation of the blue-green pigment (number 3 in Figure 2C). The yield of indigoidine could be increased by adding 2.5 mM glutamine as precursor to the reaction, as indicated by the darker blue-green color. However, the addition of 5 mM glutamine did not increase the color intensity significantly, indicating that higher indigoidine levels may interfere with the components of the cell-free lysate.

### Betalain Biosynthesis

Finally, we used the BYL system to produce betalains from the amino acid tyrosine. We investigated two overlapping pathways for the biosynthesis of (1) yellow-orange betaxanthins, which requires the monophenolase activity of the cytochrome P450 BvCYP76AD5 from *B. vulgaris* and the dioxygenase MjDOD from *M. jalapa*; and (2) the red-violet betacyanin betanin, which requires the monophenolase as well the diphenolase activity of the cytochrome P450 BvCYP76AD1_W13L from *B. vulgaris*, MjDOD, and the glucosyltransferase MjcDOPA5GT from *M. jalapa* (Figure 3A). As above, all four enzymes were first produced separately, allowing us to verify the correct size of the proteins by SDS-PAGE (Figure 3B). As anticipated, we detected bands of 56 kDa (BvCYP76AD1_W13L), 57 kDa (BvCYP76AD5), 30 kDa (MjDOD) and 57 kDa (MjcDOPA5GT). The yields of all four proteins were lower than the 29 kDa eYFP control, with particularly weak bands representing BvCYP76AD1_W13L and BvCYP76AD5 barely visible against the background of host cell proteins in the BYL.

For the synthesis of betaxanthins, BvCYP76AD5, and MjDOD were expressed alone or together using the same molar concentration of template DNA (5 nM or 2.5 nm each). The exclusive monophenolase activity of BvCYP76AD5 ensured that the L-DOPA was channeled toward the synthesis of betalamic acid and subsequently betaxanthins (Grewal et al., 2018). The reactions containing BvCYP76AD5 alone remained the same color as the non-template control (number 1 and 2 in Figure 3C). The reactions containing MjDOD alone turned slightly more yellow (number 3 in Figure 3C), indicating that the precursor L-DOPA is present in the cell-free lysate. In plants, L-DOPA is a precursor of many alkaloids, catecholamines, and melanin, which is present in many tissues (Soares et al., 2014). The co-expression of both enzymes led to the high accumulation of yellow-orange betaxanthins (number 4 in Figure 3C). Adding the precursor tyrosine (5 mM) at the beginning of the reaction increased the product levels as indicated by the significantly higher color intensity and change from yellow-orange to orange.

Similarly, the cell-free production of betanin was demonstrated by expressing BvCYP76AD1_W13L, MjDOD, and MjcDOPA5GT alone or in all possible combinations using the same molar concentrations of template DNA (5 nM). In this reaction, the monophenolase activity of BvCYP76AD1_W13L produced L-DOPA but the additional diphenolase activity converted it into *cyclo*-DOPA (Grewal et al., 2018). When all three enzymes were present, the color of the cell-free lysate turned from pale yellow to red-violet after 24 h (Figure 3D). Adding the precursor tyrosine (5 mM) at the beginning of the reaction increased the yield of betanin, as shown by the greater intensity of the red-violet color. No color development (and thus no betanin accumulation) was detected when any of the enzymes were produced alone, but the co-expression of the first two enzymes of the pathway (BvCYP76AD1_W13L and MjDOD) resulted in a yellow-orange color, indicating the formation of various betaxanthins resulting from the spontaneous reaction of betalamic acid with amino acids and the unstable, red-violet betacyanin betanidin (Figure 3A). Once again, adding the precursor tyrosine increased product yields resulting in a more intense color.

To determine the betanin content, the cell-free reactions were repeated at the 500-μL scale using the same molar concentrations of template DNA (1.73 nm each) with and without 5 mM tyrosine. The betanin concentration was determined after 24 h by UHPLC. The yield in the absence of tyrosine was 125 μg betanin per mL cell-free reaction, whereas the provision of 5 mM tyrosine increased the yield to 470 μg per mL. Higher molar concentrations of template DNA (3.33 nm each) increased the betanin yield even further to 555 μg per mL cell-free reaction. These remarkable yields demonstrate that the cell-free BYL system is not only suitable for the analysis of metabolic pathways but also for the rapid production of target molecules.

## Discussion

We have demonstrated for the first time the successful production of secondary metabolites in the cell-free BYL system. Thus far, cell-free lysates prepared from tobacco BY-2 cells have been used as a powerful screening platform for recombinant proteins in small-scale reactions of 50–100 μL and for the production of recombinant proteins that are difficult to express in living cells due to their toxicity (Havenith et al., 2017; Huck et al., 2017; unpublished data). Notably, the BYL contains active mitochondria that deliver energy for protein synthesis and can synthesize recombinant proteins with yields of up to 3 mg per mL by *in vitro* transcription–translation in batch reactions, which is ∼15 times more productive than other eukaryotic cell-free systems in batch mode (Madduri et al., 2018; Schillberg et al., 2019). Glutamate supplied to the BYL in the form of magnesium glutamate and potassium glutamate can enter the citrate cycle within the mitochondria *via* α-ketoglutarate, leading to the formation of the reducing equivalents NADH and FADH. Electrons enter the electron transport chain *via* NADH and FADH to generate ATP through oxidative phosphorylation consuming molecular oxygen (Madduri et al., 2018). Consequently, the BYL system can also be used to produce larger quantities of metabolic enzymes for analysis or for the synthesis of specific metabolites. We clearly demonstrated this application by the successful production of lycopene, indigoidine, and betalains.

Importantly, the preparation of cell-free lysates can be decoupled from recombinant protein expression and metabolite biosynthesis. Following lysate preparation, ready-to-use reaction samples can be stored at –80°C for up to 1 year. If necessary, the template DNA (plasmids or PCR products carrying the expression cassettes for the target proteins) can be added to the thawed lysate to initiate biosynthesis. The cell-free platform thus provides a flexible, on-demand tool for the analysis and optimization of metabolic pathways, and can provide sufficient quantities of secondary metabolites for various assays without the costly infrastructure required for cell-based protein production. It is therefore unsurprising that cell-free expression has already been exploited for such approaches, although these systems are typically derived from microbial cells (for review see Bogart et al., 2021). Cell-free systems have been used, for example, to characterize and optimize specific enzymes (Körfer et al., 2016; Kightlinger et al., 2018), and to mix and match combinations of enzymes to rapidly prototype and optimize metabolic pathways (Kightlinger et al., 2019; Jaroentomeechai et al., 2020; Karim et al., 2020). We selected lycopene, indigoidine and betalains as case studies for the BYL platform because they are industrially relevant molecules that can be detected and quantified using simple colorimetric methods, and there is already a large amount of appropriate comparative data from cell-based and cell-free production systems.

The major commercial source of lycopene is currently tomato fruits, although the extraction of lycopene from plants is expensive and environmentally harmful. This has prompted the transfer of the metabolic pathway to microbial production platforms such as *E. coli*, *Saccharomyces cerevisiae* and *Yarrowia lipolytica* (Li et al., 2020; Wang et al., 2020). During the last year, the productivity of these engineered biofactories has been improved significantly, mainly by engineering gene expression, improving lycopene storage capacity, optimizing fermentation processes, and combinatorial pathway engineering (Li et al., 2020). The highest lycopene titer achieved thus far is 4.2 g per L culture medium in the yeast *Y. lipolytica*, which was achieved by a combination of pathway engineering and improved fermentation strategies (Luo et al., 2020). The yield in the BYL system was significantly lower (21 μg/mL) but this is, to our knowledge, the first time lycopene has been produced in a cell-free expression system and thus provides a versatile platform for the production optimization of carotenoids and other high-value isoprenoids.

In a similar effort to improve the synthesis of indogoidine, the *S. lavendulae bpsA* gene has been transferred to the industrial production strains *Corynebacterium glutamicum* and *Rhodosporidium toruloides*. When combined with metabolic engineering and the optimization of fermentation strategies, this achieved indigoidine yields of 49 and 86 g per L, respectively (Wehrs et al., 2019; Ghiffary et al., 2021). Indigoidine has also been produced by cell-free biosynthesis in the commercial *E. coli*-based PURExpress system (Siebels et al., 2020). This system contains all necessary components of the *E. coli* transcription and translation machinery, which have been produced as recombinant proteins and combined in defined concentrations (Shimizu et al., 2001). In contrast to our study, the cell-free synthesis of indogoidine in the PURExpress system was achieved by expressing *S. lavendulae* BpsA but not *B. subtilis* Sfp, which was instead added to the reaction along with coenzyme A to enable the post-translational phosphopantetheinylation of the BpsA thiolation domain (as required for substrate shuffling). The reported yield was up to 250 μM (62 mg/L). More precise quantitative analysis was not possible due to the lack of suitable standards, which is also why we did not determine the concentration of indigoidine in our study. Nevertheless, the colorimetric assay confirmed the accumulation of large amounts of indigoidine, indicating that the BYL system can be harnessed for the rapid engineering and optimization of indigoidine biosynthesis.

The production of betalains has also been transferred to microbes by metabolic engineering. Various betaxanthins and betacyanins have been produced in *E. coli*, with yields of up to 288 mg per L (Guerrero-Rubio et al., 2019; Hou et al., 2020). Grewal et al. (2018) reported the production of betalains in the yeast *S. cerevisiae*, achieving a titer of 17 μg per mL for betanin, the most industrially relevant betalain. To our knowledge, the cell-free biosynthesis of betalains has not been reported thus far. The successful production of betaxanthins and betacyanins in the BYL system is therefore the first step toward the engineering and optimization of betalain biosynthesis in this platform. Notably, we achieved yields of up to 555 μg betanin per mL BYL solution, which is 32 times higher than previously achieved by cell-based expression in *S. cerevisiae* (Grewal et al., 2018) and thus the highest yield thus far achieved by metabolic engineering.

We have shown that the cell-free BYL system can support the production of secondary metabolites, using representatives from the carotenoids, NRPs and betalains as case studies. In the future, we will optimize the BYL system to improve the accumulation of lycopene, indigoidine and betalains by testing enzymes from alternative organisms and/or by optimizing enzyme sequences and improving reaction conditions before transferring optimized pathways to cell-based production platforms. In addition, metabolic pathways can be modified and augmented to synthesize products with improved or new properties, including xenobiotics that are not found in nature because they are unstable and/or quickly metabolized in living cells. This could provide a new source of novel active substances as drug leads. The cell-free BYL system can be scaled up inexpensively by using more BY-2 cell biomass for lysate preparation, with work underway to reach scales of 1 L and even 10 L per reaction (Schillberg and Finnern, 2021). This would facilitate the production of gram quantities of secondary metabolites, making cell-based systems ultimately unnecessary for challenging products.

## Data Availability Statement

The original contributions presented in the study are included in the article/Supplementary Material, further inquiries can be directed to the corresponding author/s.

## Author Contributions

MB and SS conceived the study and wrote the manuscript. MB, NH, and AC conducted the experiments and collected the data. All authors interpreted data, proofread, and approved the manuscript.

## Conflict of Interest

SS is member of the Scientific Advisory Board of LenioBio GmbH, which distributes the BY-2 cell-free lysate developed by Fraunhofer IME and Dow AgroSciences. The remaining authors declare that the research was conducted in the absence of any commercial or financial relationships that could be construed as a potential conflict of interest.

## Publisher’s Note

All claims expressed in this article are solely those of the authors and do not necessarily represent those of their affiliated organizations, or those of the publisher, the editors and the reviewers. Any product that may be evaluated in this article, or claim that may be made by its manufacturer, is not guaranteed or endorsed by the publisher.
