# Supplementary material for: Plant-Derived Cell-Free Biofactories for the Production of Secondary Metabolites
Source: Front Plant Sci. 2022 Jan 28;12:794999. doi: 10.3389/fpls.2021.794999 (PMC8832058; doi:10.3389/fpls.2021.794999)
Supplement: Supplementary file 1 [file Data_Sheet_1.DOCX]

Plant-derived cell-free biofactories for the production of secondary metabolites

Matthias Buntru^1^, Nils Hahnengress^1^, Alexander Croon^1^, Stefan Schillberg^1,2*^

^1^Fraunhofer Institute for Molecular Biology and Applied Ecology IME, Aachen, Germany

^2^Institute of Phytopathology, Justus Liebig University, Giessen, Germany

*** Correspondence:**Stefan Schillberg
stefan.schillberg@ime.fraunhofer.de


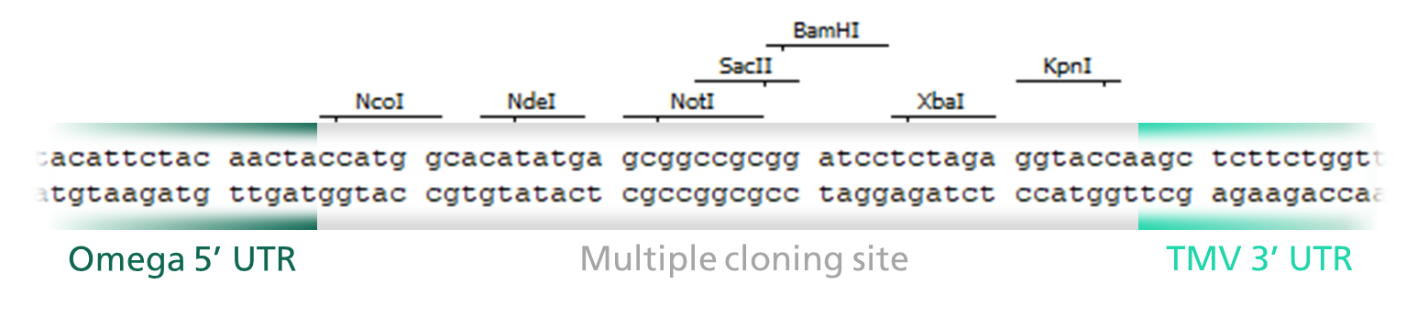


**Figure S1A.** Multiple cloning site in the pLenEx expression vector. Omega 5’ UTR, Tobacco mosaic virus 5’ untranslated region; TMV 3’ UTR, Tobacco mosaic virus 3’ untranslated region.

| **Expression cassette** | **Pathway** |
| --- | --- |
| 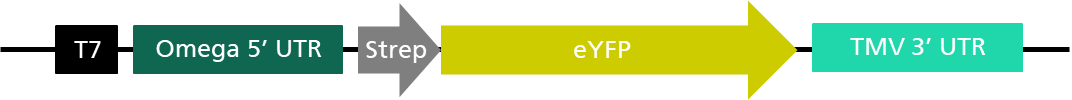 | - |
| 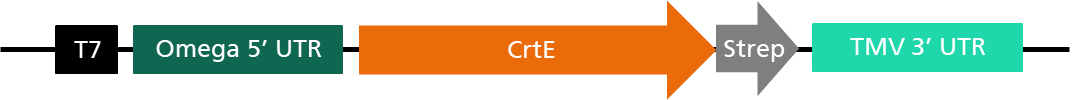 | Lycopene |
| 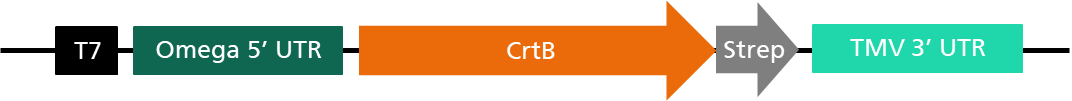 | Lycopene |
| 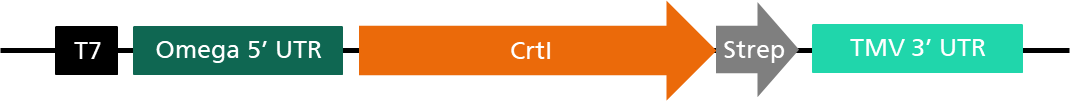 | Lycopene |
| 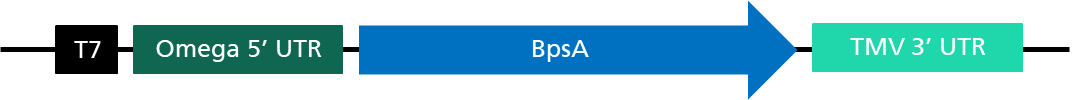 | Indigoidine |
| 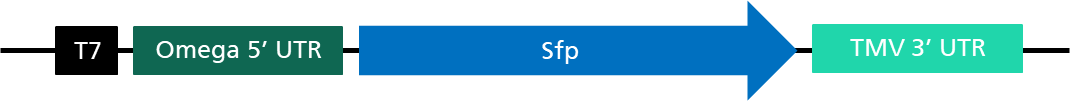 | Indigoidine |
| 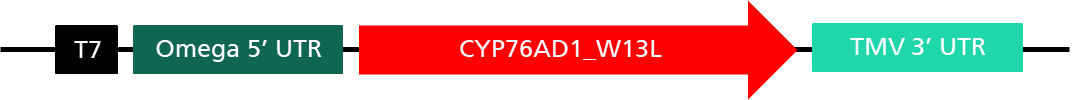 | Betalains |
| 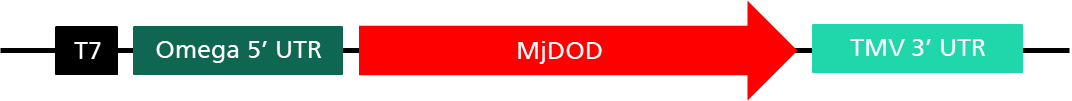 | Betalains |
| 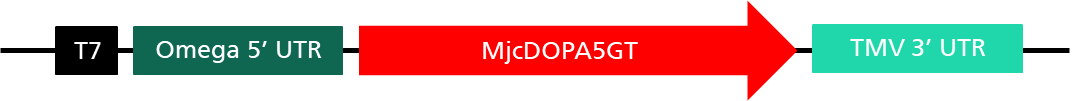 | Betalains |
| 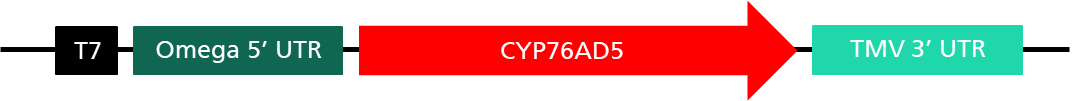 | Betalains |

**Figure S1B.** Physical map of expression cassettes used in this work (not drawn to scale). T7, T7 RNA polymerase promoter; Omega 5’ UTR, Tobacco mosaic virus 5’ untranslated region; Strep, streptavidin affinity tag sequence; eYFP, cDNA encoding for the enhanced yellow fluorescent protein from Aequorea victoria; CrtE, geranylgeranyl diphosphate synthase from Pantoea ananatis; CrtB, phytoene synthase from P. ananatis; CrtI, phytoene desaturase from P. ananatis; BpsA, blue pigment synthetase A from Streptomyces lavendulae; Sfp, 4’-phosphopantetheinyl transferase from Bacillus subtilis; CYP76AD1_W13L, monophenolase/diphenolase from Beta vulgaris; MjDOD, DOPA-4,5-dioxygenase from Mirabilis jalapa; MjcDOPA5GT, cyclo-DOPA glucosyltransferase from M. jalapa; CYP76AD5, monophenolase from Beta vulgaris; TMV 3’ UTR, Tobacco mosaic virus 3’ untranslated region.
